# Nephrotic Syndrome in a Child Suffering from Tetralogy of Fallot: A Rare Association

**DOI:** 10.1155/2015/128409

**Published:** 2015-08-03

**Authors:** Pépé Mfutu Ekulu, Orly Kazadi-wa-Kazadi, Paul Kabuyi Lumbala, Michel Ntetani Aloni

**Affiliations:** ^1^Division of Paediatric Haemato-Oncology and Nephrology, Department of Paediatrics, University Hospital of Kinshasa, Faculty of Medicine, University of Kinshasa, Kinshasa, Congo; ^2^Division of Cardiology, Department of Paediatrics, University Hospital of Kinshasa, Faculty of Medicine, University of Kinshasa, Kinshasa, Congo

## Abstract

Nephrotic syndrome is an uncommon complication of tetralogy of Fallot and has been rarely reported in pediatric population. We describe a 4-year-old female Congolese child who was referred for investigation for persistent dyspnea, edema, and cyanosis and nephrotic range proteinuria. Our patient presented with a tetralogy of Fallot and nephrotic syndrome. *Conclusion*. This case reminds us that children with tetralogy of Fallot may develop nephrotic proteinuria.

## 1. Introduction

Glomerular dysfunction can be found in cyanotic congenital heart disease (CHD) especially in older children and adults, being associated occasionally with proteinuria and microalbuminuria [1, 2]. The risk of developing renal impairment is particularly high in cyanotic patients particularly in patients with long-standing cyanotic CHD [1, 2]. However, nephrotic syndrome (NS) is an uncommon complication of cyanotic congenital heart disease and is rarely reported. This complication has not been documented in Congolese children.

## 2. Case Report

A 4-year-old girl with edema, dyspnea, and cyanosis was referred from Butembo at the East of the Democratic Republic of Congo (DRC) to a facility renal and cardiology investigations in Department of Pediatrics of University Hospital of Kinshasa, Kinshasa, DRC. The history of present illness dates back to about 13 months characterized by progressive cough, dyspnea, and orthopnea. Physical examination revealed respiratory distress with edema and episodes of squatting. She was cyanosed with finger and toes clubbing. Apex beat was at the fifth intercostal space anterior axillary line. Both heart sounds were noted with a systolic thrill and loud systolic ejection murmur grade 3. The blood pressure was 150/110 mmHg, and oximetry was 63%.

A complete blood count showed hemoglobin 23.2 g/dL, total proteins 45 g/L, and albumin 22 g/L. Dipstick urinalysis was 3 + while the 24-hour urinary protein was 154 mg/kg. Creatinine was 37 *μ*mol/L, urea was 3.8 mmol/L, cholesterol was 4.3 mmol/L, and HDL was 1.3 mmol/L. HIV and hepatitis serology were negative. Anti-streptolysin O (ASLO) was <200 IU.

**Figure 1:**
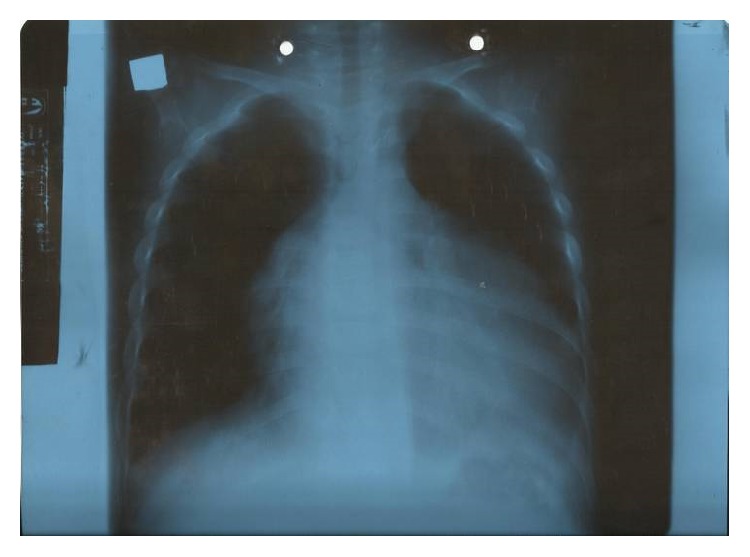
Plain film shows a “boot shaped” heart with an upturned cardiac apex due to right ventricular hypertrophy and concave pulmonary arterial segment.

X-ray revealed “boot shaped" heart with an upturned cardiac apex (Figure 1). Echocardiography revealed tetralogy of Fallot with hypoplastic pulmonary artery and biventricular dysfunction. Cardiac catheterization was not performed due to technical reasons. A diagnosis of Fallot's tetralogy and NS was established. Renal biopsy was contraindicated because of the deteriorating renal condition and cardiac status.

During her hospitalization, the child received specific treatment for her blood hypertension, associated furosemide (1 mg/kg/dose, every six hours), Propranolol (2 mg/kg, every six hours), and Enalapril (0.4 mg/Kg, every twelve hours). For her NS, the child received prednisone (60 mg/m^2^/day during 30 days) for the first phase and diet.

Initial biologic and clinical improvement was observed with steroid and Enalapril therapies. Edema regressed with treatment. The 24-hour urinary protein decreased from 154 mg/kg to 6.7 mg/kg and creatinine remained stable. Death occurred 41 days after admission to an array of sudden hypoxic tet spells. She was stable enough to be discharged after six weeks on admission.

## 3. Discussion

Nephropathy among patients is an important complication of tetralogy of Fallot [1, 2]. The incidence and prevalence are unknown and epidemiological data are rare in Africa. In Central Africa, this affection was not previously noted. Our observation is the first description in our population. Only three cases in pediatric population could be found in Nigeria with the use of available computer-assisted medical literature search programs [1, 2]. The situation is probably due to the lack of the evaluation of renal function in highly resource-scarce settings and nephrologist pediatrician [3]. At the same time, it is probable that many children with cyanotic CHD die before the development of NS in our midst.

We have reviewed the literature on cyanotic CHD and NS (Table 1). The development of NS associated with cyanotic CHD remains unclear. However, some aetiopathogenetic mechanisms have been suggested for the development of nephropathy. The patients with cyanotic CHD are exposed to chronic hypoxia. The risk of developing glomerular lesions rose sharply during the second decade of life if the cyanosis remains unchanged for more than ten years [4, 5]. Hyperviscosity due to polycythemia may induce an angiogenic increase in the glomerular capillary beds, in turn leading to glomerulomegaly. Glomerulomegaly is a consequence of the hyperperfusion of glomeruli associated with the chronic hypoxia and the increased hydrostatic pressure in the capillary wall. This situation is a causative factor of the deterioration and the decline of renal function, in the condition of polycythemia. Furthermore, the failure of a compensatory mechanism to respond to reduced RPF by hyperfiltration may be accompanied by the development and progression of microalbuminuria and proteinuria [4–6]. The pathogenesis and development of nephrotic proteinuria range are the result of these combined mechanisms. Although we could not perform the renal biopsy, the nephrotic range proteinuria is probably a consequence of focal and segmental glomerulosclerosis.

Our patient presented late. This case revealed the problem of early diagnosis, regular follow-up, and early detection of complications in African cyanotic CHD patients especially in case of Fallot's tetralogy during antenatal or neonatal period. It is known that untreated cardiac malformations in patient with Fallot's tetralogy have high likelihood of progression to glomerular damage [4–6]. In our context, diagnosis and management were generally delayed.

It is worth considering the use of ACE-I when nephropathy accompanies cyanotic CHD. In a previous study, Enalapril apparently reduced the urinary protein excretion in 80% of patients [7]. In our case, proteinuria decreased from 154 mg/kg/24 h to 6.7 mg/kg/24 h.

This case reminds us that children with tetralogy of Fallot may develop NS. This case report is pointing out the problem encountered in the early diagnosis and management of cyanotic CHD in resource-limited settings as in DRC. Considering the paucity of facilities available for medical and surgical management of Fallot's tetralogy in our midst, we recommend early detection of this congenital heart disease and regular renal screening of patients and thus allow, at least at this stage, the initiation of ACE-I. However, it has to be stated that, in countries such as the DRC, early corrective cardiac surgery should be the first choice particularly in patients who otherwise present severe complications of their cyanotic CHD and reduce the risk of the development of chronic renal failure.

## Figures and Tables

**Table 1:** Results of the literature review of cyanotic CHD associated with nephrotic syndrome in African children.

Source	Our case	Adedoyin and Afolabi [2]	Ogunkunle et al. [1]	Ogunkunle et al. [1]
Country	Kinshasa, DRC	Ilorin, Nigeria	Ibadan, Nigeria	Ibadan, Nigeria
Year of description	2015	2006	2008	2008
Age (years)	4	9	12	7
Gender	Female	Male	Female	Male
Cyanotic CHD^*∗*^	Tetralogy of Fallot	Truncus arteriosus	Tetralogy of Fallot	Tricuspid atresia

^*∗*^CHD: congenital heart disease; DRC: Democratic Republic of Congo.
